# Relationships Between the Therapeutic Alliance and Reactions to Artistic Experience With Art Materials in an Art Therapy Simulation

**DOI:** 10.3389/fpsyg.2021.560957

**Published:** 2021-07-14

**Authors:** Inbal Gazit, Sharon Snir, Dafna Regev, Michal Bat Or

**Affiliations:** ^1^The School of Creative Arts Therapies, University of Haifa, Haifa, Israel; ^2^Department of Art Therapy, Tel Hai College, Upper Galilee, Israel

**Keywords:** triangular relationship, working alliance, reactions to artistic experience, art therapy simulation, therapeutic relationship

## Abstract

In art therapy, art-making plays an important role in the therapeutic relationship. To better understand the triangular relationship between the art therapist, the client and the artwork, this study investigated the association between the therapeutic alliance and reactions to artistic experiences with art materials in an art therapy simulation. The simulation consisted of a series of 6–8 sessions in which art therapy students were divided into teams composed of a permanent observer (art therapist) and creator (client). The client's role was to self-explore through art- making, and the art therapist's role was to accompany the client. Thirty-four students, all women, who played the art therapist role, and 37 students (one male) who played the client participated in the study. Of these participants, there were 24 pairs where both participants filled out all the questionnaires. A short version of the Working Alliance Inventory (WAI) was completed by the clients and the art therapists on the second session (T1) and on the penultimate session (T2). The clients also completed the Art-Based Intervention Questionnaire (ABI) at T2. Significant positive correlations were found between indices of the WAI for the art therapist and the client and the clients' reactions to the artistic experience with art materials on the ABI. The evaluation of the emotional bond between the art therapist and the client at the start of the simulation significantly predicted the client's reactions to the artistic experience with art materials at the end of the simulation and explained 45.4% of the variance for this variable. These findings highlight factors related to the development and influence of the therapeutic alliance, as well as the role of the artistic experience in art therapy and lay the groundwork for further research.

## Introduction

The relationship between therapist and client is considered crucial to development, personal growth, therapeutic change, and positive therapeutic outcomes (Gullo et al., 2012). Work with art materials in art therapy contributes to the dyadic therapist-client relationship (Robbins, 2001) and forms what is known as the triangular relationship between the art therapist, the client and the artwork (Schaverien, 2000). This study explored the associations between the side of the triangle characterizing client-art therapist relations, and the side related to client-artwork relations.

The relationship between the therapist and the client, which is often referred to as the therapeutic alliance (Doran, 2016), is a cornerstone of the psychotherapeutic process (Zilcha-Mano et al., 2014) and is generally viewed as the collaborative component of the therapeutic relationship. In the literature, it is defined as consisting of three components: agreement on the goals of therapy (Goal), collaboration on tasks that help to achieve these goals (Task), and the development of an emotional bond between therapist and client (Bond) (Bordin, 1979). The alliance is considered to contribute in a significant way to change over the course of the psychotherapeutic process (Murphy and Hutton, 2018; Welmers-van de Poll et al., 2018). The strength of the therapeutic alliance has been shown to be a major predictor of high psychological functioning, can reduce the severity of negative symptoms, and promote a better quality of life (e.g., Xu and Tracey, 2015).

In art therapy, art materials are the prime constituents of therapeutic interventions and can be considered to add a third dimension to the therapeutic alliance to form a triangular relationship between the art therapist, the client and the artwork (Schaverien, 2000; Moon, 2010). One of the key theoretical assumptions in art therapy is that artwork, which expresses the inner world of the client (Hilbuch et al., 2016), may also reflect or deepen the relationship between the art therapist and the client (Robbins, 2001). Another crucial theoretical assumption is that the art therapist serves as an active partner in the process of art-making by providing the materials, and enabling a safe space in which clients can make associations, revisit emotions from the past, explore, and discover new things about themselves through their artistic experiences (Dalley et al., 1987; Wood, 1990; Hilbuch et al., 2016; Snir et al., 2017). These two theoretical assumptions suggest a connection between the client's relationship with the art therapist and his or her relationship with the artwork.

This connection between the two sides of the triangle may also stem from the role attributed to object relations on each of these sides. In terms of the client's relationship with the therapist, as often measured by the WAI (Working Alliance Inventory) (Horvath and Greenberg, 1989), there is ample evidence that clients' object relations and the quality of their interpersonal relationships (Ollila et al., 2016) play an important role in the development of the therapeutic alliance, such that positive internal representations are related to the formation of a strong and stable working alliance (Mikulincer et al., 2013; Sanders et al., 2014), whereas negative internal representations disrupt the development of the alliance (Bernecker et al., 2014). In terms of the client-artwork relations, theorists suggest that the clients' object relations are reflected in their responses to the artistic experience with the art materials before, during, and after art-making, as well as to the product itself (Robbins, 2001; Sanders et al., 2014; Hilbuch et al., 2016). This is consistent with a previous study (Snir et al., 2017) on the relationships between attachment dimensions of anxiety and avoidance and reactions to using art materials in 409 non-client volunteers. These participants engaged in art-making and then completed the ECR (Experience in Close Relationship Questionnaire) (Brennan et al., 1998) which measures attachment, and the ABI (Art Based Intervention Questionnaire) (Snir and Regev, 2013), which measures clients' attitudes toward creating and using art materials. The findings indicated that the higher the score on the avoidance dimension, the more negative the individual's response to the art materials.

Some support for the existence of an association between client-art therapist and client-artwork relationships emerged from a preliminary correlative study in which 51 participants filled out the Client Attachment to Therapist Scale (CATS) (Mallinckrodt et al., 1995) and the ABI at the end of a simulated therapeutic session. The results suggested that the higher the client's secure attachment to the art therapist in the simulation, the more positive the experience of working with the art materials. As in the study above, the greater the avoidance reported in the relationship with the art therapist during the simulation, the more negative the experience of working with art materials (Corem et al., 2015).

The present simulation study sought to empirically test the association between the client-art therapist side in the art therapy triangular relationship as assessed by the therapeutic alliance, and the client-artwork side, as assessed by reactions to the artistic experience with art materials. Previous research has shown that the use of simulation is a valid way to examine different treatment-related phenomena and that the results are consistent with the findings reported in field studies (e.g., Cooper, 2012; Abramowitz et al., 2014). We hypothesized that there would be a positive correlation between the three components of therapeutic alliance (Goal, Task, and Bond), and clients' reactions to the artistic experience with art materials in a classroom simulation.

## Method

### Participants

The participants were second-year art therapy students at the University of Haifa who were all enrolled in a course entitled “Advanced Concepts in Art Therapy.” Of these 96 women and four men (forming a total of *48* dyads) who were taking this course during the 2 years of data collection, 70 women and one man agreed to participate in the study. The students were randomly divided into permanent creator (who played the client role; henceforth “clients”) and observer (who played the art therapist role; henceforth “art therapists”) teams by the course instructor, while deliberately avoiding putting good friends together. Of the total number of participants, 34 played the art therapist role, and ranged in age from 26 to 53 (*M* = 34.67, *SD* = 6.91); 37 played the client role, and ranged in age from 24 to 58 (*M* = 34.12, *SD* = 7.73). Data collection took place in two repeated measurements during the simulation, and because participants had the right to decline to participate in each measurement separately, the number of participants in each was different. At T1, 29 art therapists and 30 clients (78.4 and 81.1%, respectively) completed the questionnaires, and at T2, 31 art therapists and 36 clients (83.8 and 97.3%, respectively). There were only 24 dyads where both participants completed all the questionnaires on both measurements.

### Tools

#### Working Alliance Inventory

The therapeutic alliance was measured using the short client and the therapist versions of the WAI (Horvath and Greenberg, 1989; Tracey and Kokotovic, 1989). This questionnaire is made up of 12 Likert-type items rated on a 7-point Likert scale ranging from 1 = “never” to 7 = “always.” The questionnaire assesses three dimensions: the extent of agreement between the therapist and the client about the goals of therapy (Goal component), the extent of agreement between them on how to achieve these goals (Task component) and the emotional bonding between the therapist and the client (Bond component). This questionnaire is one of the most commonly used instruments for evaluating the therapeutic alliance (e.g., Doran, 2016). Studies have reported high reliability. For example, in a study on the association between treatment outcomes in BRT vs. CBT, the internal reliability range across time points was 0.88–0.94 for clients and 0.83–0.93 for therapists (Zilcha-Mano et al., 2019). In another study on the WAI, the internal reliability at four time points ranged from 0.92 to 0.95 (Zilcha-Mano et al., 2014). The WAI was reported to evidence convergent and discriminant validity with other self-report measures of interpersonal relationships and other alliance measures, as well as with outcomes (e.g., Alexander and Luborsky, 1986; Hatcher and Gillaspy, 2006). For purposes of the present study, the questionnaire was adapted to the simulation of art therapy; for example: “___and I collaborate on setting the goals for my artistic self-exploration.” The questionnaire was filled in by both the art therapist and the client at two time points during the simulation. The Cronbach's alphas in this study ranged from α = 0.50 to α = 0.88. In addition to using the original scales, a general therapeutic alliance score (General WAI) based on the average of the three components was also calculated for each art therapist and each client, on each of the two measurements separately.

#### Art-Based Intervention Questionnaire

The self-report ABI (Snir and Regev, 2013) is composed of 41 Likert-type items that examine the participants' artistic experiences when using art materials. The clients were asked to rate each statement on a scale anchored at “very wrong” (1) to “very true” (7) to indicate the extent to which it described their experiences during the artwork in the session that had taken place just prior to the administration of the questionnaire. The items tapped four categories, under 10 sub-scales as follows: (a) feelings and thoughts before the artistic process, which included the sub-scales of positive excitement (e.g., “I was eager to begin the creative task”), confidence (e.g., “I had several ideas about what I wanted to make”), and aversion (e.g., “I was upset about getting dirty”; (b) feelings and thoughts during the artistic process, which included the pleasantness and therapeutic value (e.g., “Working on my art project released the tension I'd been feeling”), competence (e.g., “I knew exactly how to handle the materials”), difficulty in carrying out the artistic task (e.g., “I had a difficult time executing my ideas”), and playfulness (e.g., “I felt I was playing with the materials”) sub-scales; (c) attitudes toward the artistic product, one subscale (e.g., “I was excited to see what I had made”); and (d) attitudes toward the material, as captured by the meaningful (e.g., “ powerful material”) and pleasant material (e.g., “The material had a soothing effect”) sub-scales. In this study we used only 33 items from two out of the four questionnaire scales that were reported to be the most reliable by the questionnaire authors (Corem et al., 2015; Snir et al., 2017): Feeling and thoughts during the artistic process (Process component), and Attitude toward the product (Product component). This questionnaire was filled in by the clients at T2. For each client, a score was calculated for each of the two scales (Process component and Product component) as well as a general score for all questionnaire items (General-ABI). The ABI subscales were found to be positively correlated with the Session Evaluation Questionnaire (Stiles et al., 1994), and with relational measurements such as secure attachment with the therapist (Corem et al., 2015), and the attachment dimensions of anxiety and avoidance (Snir et al., 2017). The authors reported good reliability of the subscales, ranging from 0.63 to 0.86 (Snir and Regev, 2013). The Cronbach's alphas in the current study ranged from α = 0.63 to α = 0.92.

### Procedure and Ethical Considerations

This correlative exploratory study is part of a larger project exploring the triangular relationship in art therapy. It took place during an undergraduate art therapy course at the School of Creative Arts Therapies at the University of Haifa from 2015 to 2017. The clients' role was to explore their inner selves through art materials, during six or seven weekly sessions (the number of sessions varied according to the number of weekly sessions each semester). The art therapists were asked to accompany the clients in their exploration in a way that best suited the clients' needs. The data for the study were collected during the second session (T1), and at the penultimate session in each cycle (T2); i.e., sessions 2 and 6 in the 7- session simulation, or sessions 2 and 5 in the 6- session simulation. All the students in the course were invited to take part in the study, with no penalty for refusing. The questionnaires were filled out at the end of the lesson by the participating students immediately after the simulation session, and were immediately placed in sealed envelopes, keeping the art therapists' questionnaires together with their clients' questionnaires. Identification numbers were written on the envelopes, which the students chose themselves, to allow the researchers to associate information from the repeated measurements. This study was approved by the Ethics Committee of the Faculty of Welfare and Health Sciences at the University of Haifa, and complied with all regulations on ethics in human research.

### Statistical Analysis

A preliminary analysis tested for a normal distribution using the Shapiro-Wilk test. Pearson correlations tested for associations between the WAI subscales. Repeated measures ANOVAs were used to examine changes in WAI scores between times and groups. The hypotheses were tested using Pearson correlations, and standard multiple regression analyses, with SPSS software. Due to the many comparisons, a Bonferroni correction was implemented.

## Results

### Preliminary Analysis

A preliminary Shapiro-Wilk test revealed that most of the scales were normally distributed; therefore parametric tests were performed. Calculation of the scale means (see Table 1) showed that the alliance score means were *M* = 4.77 (*n* = 29) and *M* = 5.36 (*n* = 31) for the art therapists, and *M* = 4.69 (*n* = 30) and *M* = 5.38 (*n* = 36) for the clients, indicating the overall positive quality of the alliance at T1 and T2.

**Table 1:** Summary of means, standard deviations, and minimum and maximum for scores on WAI and ABI.

	**M**	**SD**	**MAX**	**MIN**
**WAI**				
Art therapist T1, *n* = 29	4.77	0.91	6.83	2.95
Art therapist T2, *n* = 31	5.36	0.74	6.80	3.76
Client T1, *n* = 30	4.69	1.07	6.44	2.10
Client T2, *n* = 36	5.38	1.00	7	2.09
**ABI, *n* = 37**				
Process	5.52	0.82	6.68	2.09
Product	5.46	1.19	7	1.17
General-ABI	5.50	0.78	6.75	1.89

The means for the ABI scales also showed that the clients reported a positive overall response to their artistic experience with the art materials, with a General-ABI *M* = 5.5 (*n* = 37).

As depicted in Table 2, positive moderate to high correlations were found between the WAI sub-scales for the clients' responses at both T1 and T2. For the art therapists' responses, these correlations were mostly moderate.

**Table 2:** Pearson's correlations between the therapeutic alliance sub-scales in art therapists and clients.

	**Art therapists**			**Clients**		
	**Goals**	**Task**	**Bond**	**Goals**	**Task**	**Bond**
Goals		*r* (T2) = 0.46 *p* = 0.010	*r* (T2) = 0.32 *p* = 0.075		*r* (T2) = 0.89 *p* = 0.000	*r* (T2) = 0.78 *p* = 0.000
Task	*r* (T1) = 0.48 *p* = 0.009		*r* (T2) = 0.68 *p* = 0.000	*r* (T1) = 0.69 *p* = 0.000		*r* (T2) = 0.87 *p* = 0.000
Bond	*r* (T1) = 0.45 *p* = 0.013	*r* (T1) = 0.61 *p* = 0.000		*r* (T1) = 0.63 *p* = 0.000	*r* (T1) = 0.67 *p* = 0.000	

*Art therapists: T1 n = 29, T2 n = 31; Client: T1 n = 30, T2 n = 36*.

**Figure 1:**
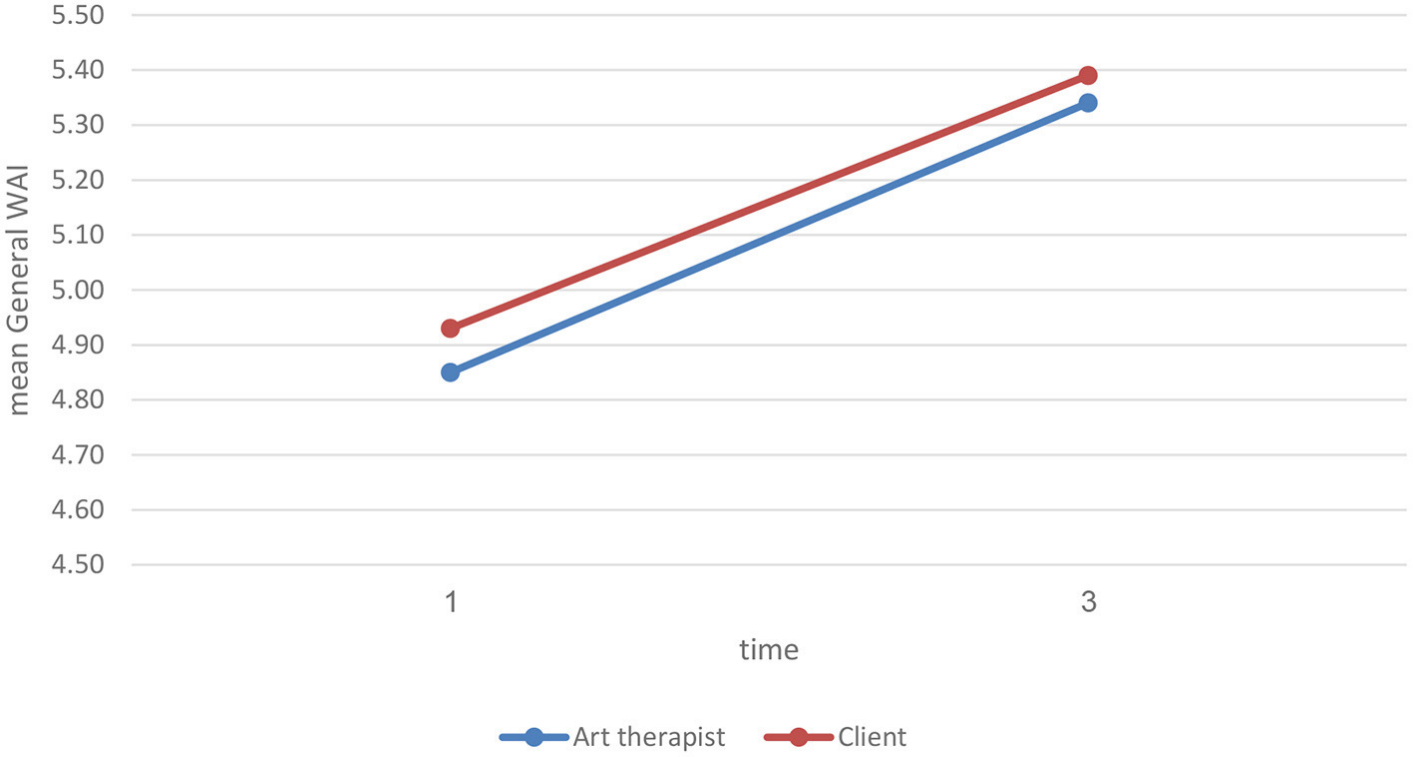
General WAI scores at T1 and T2.

We implemented a repeated measures ANOVA to examine changes in WAI scores at T1 vs. T2, and potential differences between art therapists and clients. This analysis was run solely for those pairs for whom we had no missing data (N = 24). The effect of time was significant (*p* = 0.0005). A paired *T*-test showed a significant time effect in both art therapists and clients (art therapists: at T1 4.85 ± 0.89 vs. at T2 5.34 ± 0.74, clients: at T1 4.93 ± 0.98 vs. at T2 5.39 ± 1.13, mean ± std). As depicted in Figure 1, art therapists' and clients' WAI scores were significantly higher at T2 than at T1. The effect of group was not significant (*p* = 0.7248); in other words, the art therapists and the clients were consistent in their assessment of their therapeutic alliance.

Finally, we examined whether there were significant differences in the General WAI assessment across measurements, to determine whether there was a change in the quality of the alliance. *T*-tests for paired samples indicated a significant difference between T1 and T2 for the art therapists [*T*(26) = −3.136, *p* < 0.005], who reported a higher alliance quality at T2 (*M* = 5.356, *SD* = 0.772) than at T1 (*M* = 4.837, *SD* = 0.869). Similarly, there was a significant difference in the same direction between T1 and T2 for the clients [*T*(28) = −4.145, *p* < 0.005], who reported a higher alliance quality at T2 (*M* = 5.398, *SD* = 1.08) than at T1 (*M* = 4.749, *SD* = 1.021). That is, both the art therapists and the clients reported a higher General WAI quality as the sessions progressed.

### Associations Between the Therapeutic Alliance and Reactions to the Artistic Experience With Art Materials

Pearson correlations between the therapeutic alliance quality as reported by the art therapists and the, and the clients' responses to the artistic experience with art materials showed (after the Bonferroni correction) that for the clients, there were significant high positive correlations between almost all components of the alliance at T2 (*n* = 36), and their scores for reactions to the Product, Process, and General-ABI (Table 3). There were also positive correlations between all components of the alliance at T1 (*n* = 30), and their scores for reactions to the Product. Their score for the Bond component at T1 was positively correlated to all ABI scores. Hence, for most of the correlations, the stronger the alliance, the more positive the clients' response to the artistic experience.

**Table 3:** Correlations between the therapeutic alliance components (WAI) and client reactions to the artistic experience (ABI).

**variables**			**Product**	**Process**	**General ABI**
Clients	T1 *n* = 30	General WAI	0.62 *p* = 0.000	0.30 *p* = 0.104	0.43 *p* = 0.018
		Goals	0.59 *p* = 0.001	0.22 *p* = 0.238	0.35 *p* = 0.055
		Task	0.55 *p* = 0.002	0.17 *p* = 0.370	0.30 *p* = 0.108
		Bond	0.51 *p* = 0.004	0.47 *p* = 0.009	0.53 *p* = 0.002
	T2 *n* = 36	General WAI	0.58 *p* = 0.000	0.49 *p* = 0.002	0.59 *p* = 0.000
		Goals	0.53 *p* = 0.001	0.40 *p* = 0.015	0.50 *p* = 0.002
		Task	0.58 *p* = 0.000	0.46 *p* = 0.005	0.56 *p* = 0.000
		Bond	0.53 *p* = 0.001	0.58 *p* = 0.000	0.65 *p* = 0.000
Art therapists	T1 *n* = 29	General WAI	0.10 *p* = 0.592	0.31 *p* = 0.097	0.29 *p* = 0.130
		Goals	0.16 *p* = 0.398	0.35 *p* = 0.066	0.33 *p* = 0.077
		Task	0.20 *p* = 0.309	0.37 *p* = 0.049	0.36 *p* = 0.054
		Bond	−0.14 *p* = 0.557	0.02 *p* = 0.933	−0.03 *p* = 0.863
	T2 *n* = 31	General WAI	0.41 *p* = 0.022	0.43 *p* = 0.016	0.47 *p* = 0.007
		Goals	0.47 *p* = 0.008	0.43 *p* = 0.017	0.49 *p* = 0.005
		Task	0.25 *p* = 0.166	0.35 *p* = 0.056	0.36 *p* = 0.047
		Bond	0.22 *p* = 0.236	0.22 *p* = 0.239	0.24 *p* = 0.186

When the therapeutic alliance was measured from the art therapists' perspective, there were only a few marginally significant positive correlations after the Bonferroni correction between the General WAI score and the Goals sub-scale, and the ABI scales at T2 (*n* = 31).

### Further Analyses

In addition, we conducted a standard multiple regression analysis to predict the General-ABI score of the clients at T2, using the three components of the alliance (Goals, Task, and Bond) as assessed by the art therapists and the clients at T1, after we ruled out possible multi-collinearity among the predictive variables; namely, the different therapeutic alliance sub-scales. The regression model was significant [*F*_(6, 19)_ = 4.471, *p* = 0.006] and accounted for 45.4% of the variance in the General-ABI score. The significant predictors of the regression were the Bond component according to the clients (β = 0.567, SE = 0.57, *p* = 0.014) and the Bond component according to the art therapists (β = −0.480, SE = 0.18, *p* = 0.025). That is, the assessment of the Bond component of the therapeutic alliance by the art therapists and the clients as measured at T1 explained almost half of the variance in the overall measure of the response to the artistic experience at T2.

## Discussion

The present study examined the associations between the therapeutic alliance and clients' responses to their artistic experiences with art materials during an art therapy simulation. The hypothesis of a positive association between the therapeutic alliance and the client's responses to artistic experiences with art materials was largely confirmed.

### Preliminary Analysis–The Therapeutic Alliance

The findings indicated consistency between the art therapists' assessment and the clients' assessment of the nature of the therapeutic alliance. Similarly, literature in the field of psychotherapy has consistently reported positive correlations between therapists' ratings and clients' ratings of the alliance (Shick Tryon et al., 2007; Hawley and Garland, 2008). There were also significant differences between the first measurement and the second measurement of the therapeutic alliance, both among the art therapists and the clients, indicating that the therapeutic alliance strengthened during the simulation. This finding is consistent with studies showing that the therapeutic alliance grows stronger as therapy progresses (e.g., Goldman and Anderson, 2007; Stiles and Goldsmith, 2010). Hence, although this study was conducted during a short-term art therapy simulation, the art therapists and the clients were able to form a consistent therapeutic alliance, which both of them evaluated in a similar way, and which was strengthened from the beginning of the simulation to the end. These findings are partially consistent with psychotherapy studies (e.g., Hawley and Garland, 2008; Bucci et al., 2016), thus lending weight to the efficacy of studies conducted during treatment simulations.

### Correlations Between the Therapeutic Alliance and the Clients' Response to the Artwork

In line with the hypotheses, there were significant positive correlations between the measures of the therapeutic alliance and measures of the clients' responses to the artistic experience. When the therapeutic alliance was evaluated by the clients, positive strong correlations were found between most of the alliance components, as measured at T1 and T2, and the clients' attitude toward the product, response to the process, and general score on the ABI. The more positive the therapeutic alliance as perceived by the clients, the more positive their response to the artistic experience and attitude toward the product. The art therapists' points of view with respect to the alliance in general, and the alliance Goal component were also associated with the clients' response to the artistic process and the general score for the artistic experience at T2.

These findings partially support the central assumption that there is a positive association between the art therapist-client relationship and the client-artwork relationship in art therapy. These findings are in line with another study conducted during an art therapy simulation that used the same self-report questionnaire (ABI) (Snir and Regev, 2013), which found that the higher the level of attachment security of the clients to their art therapists, the more positively they reacted to the artistic experience on most sub-scales, including feelings before the artwork, during the artwork, as well as the attitudes toward the product and the material (Corem et al., 2015).

Because this was a correlative study, no causality can be inferred from the findings. Hence, these associations may be due to a third factor affecting each of the variables, such as the clients' internal representations which could have been expressed both in relation to the art therapist (Robbins, 2001) and in relation to the artwork (Schaverien, 2000; Hilbuch et al., 2016). The results may also be explained by the theoretical assumption that the response to the artwork is an expression of the client's perception of the therapeutic relationship within which it took place. Hence, the clients may have realized over time that the art materials that helped them to express and understand themselves were part of what the art therapist could provide them when accompanying (Robbins, 2001). However, given the order in which the variables were measured in this study, where the therapeutic alliance measured at the beginning of the simulation was associated with the response to the artistic experience at the end of the simulation, it can be assumed that a stronger therapeutic alliance formed at the beginning of the simulation resulted in a more positive client response to the artwork experience at the end.

Accordingly, when taking into account all the components of the therapeutic alliance as measured on the first measurement, the Bond component, as reported by both the clients and art therapists, was found to be a significant predictor explaining 45.4% of the variance in the clients' response to the artistic experience, as measured at T2. This finding is interesting in light of the fact that the correlation tests did not identify any correlations between the art therapists' Bond component and the art therapists' reactions to the artistic process. This finding suggests that the association between the Bond component of the alliance on the part of the art therapist and the response to the artwork of the client may vary when all the components of the alliance are taken into account from the perspective of the client as well as that of the art therapist. This finding may imply that at different levels of the clients' therapeutic alliance, the association between the art therapists' perception of the Bond component and the clients' response to their artwork may be different. We were unable to conduct further analyses of the clients' therapeutic bond as a mediator, because of the small sample size, which should be explored in future studies.

The correlations between the therapeutic alliance as assessed by the clients and the art therapists as to their agreement on the goals of the simulation at T2, and the attitude toward the artistic product, showed that the attitude toward the product was a significant component of the clients' response to the artistic experience. This finding lends weight to the theoretical assumption of the importance of attitude toward the artistic product, which serves as a way to relate to the therapeutic outcome by illustrating parts of the client's inner world, which can convey significant aspects of the evolving relationship between the client and the art therapist (Hilbuch et al., 2016).

### Implications for Research

The findings point to the potential association between the therapeutic alliance in particular, and the therapist-client relationship in general, and the creative space present in art therapy. This central theoretical assumption has not been investigated to date, which makes this study one of the first in the field of research. It may thus enrich theory on the relationship triangle in art therapy. The findings and the limitations of this study can be a starting point for further research on the subject.

### Limitations and Suggestions for Further Research

This study was conducted on a relatively small sample of students in an art therapy simulation. Some of the data were missing, which further reduced the dataset and thus precluded more complex statistical analyses. Therefore, the findings cannot be generalized and only provide a first glance into the association between the therapeutic alliance and the response to art-making within the art therapist-client relationship. Examining the research question in the context of simulation in which there is no therapeutic goal, and less dependency than in a real-world therapeutic relationship further reduces the ability to generalize from the findings. The sample, which was not large in the first place, became even smaller in the regressions, and did not allow us to run follow-up tests. As such, the ability to further explore and generalize from the findings was constrained.

Future long-term research in the clinical field, using a larger sample and gender diversity is essential to support these claims. The fact that the study was based on self-report questionnaires, which are sensitive to social desirability and situational biases, may also limit its validity. Interviews with art therapists and clients about their experiences of art-making as part of an evolving alliance could help better understand the significance of this relationship.

## Data Availability Statement

The raw data supporting the conclusions of this article will be made available by the authors, without undue reservation.

## Ethics Statement

The studies involving human participants were reviewed and approved by Ethics Committee of the Faculty of Welfare and Health Sciences at the University of Haifa. The patients/participants provided their written informed consent to participate in this study.

## Author Contributions

This research was part of a larger project that was initiated, planned, coordinated, and conducted by MB, DR, and SS. IG wrote her MA thesis on part of the data, collected the data, conducted the statistical analyses, and wrote the manuscript. SS and DR supervised the writing and took part in its final stages. All authors contributed to the article and approved the submitted version.

## Conflict of Interest

The authors declare that the research was conducted in the absence of any commercial or financial relationships that could be construed as a potential conflict of interest.
